# A smartphone app intervention for adult cannabis users wanting to quit or reduce their use: a pilot evaluation

**DOI:** 10.1186/s42238-019-0009-6

**Published:** 2019-08-16

**Authors:** Lucy Albertella, Lisa Gibson, Sally Rooke, Melissa M. Norberg, Jan Copeland

**Affiliations:** 1 National Cannabis Prevention and Information Centre, UNSW Sydney, Kensington, NSW Australia; 2 Centre for Emotional Health, Department of Psychology, Macquarie University, North Ryde, NSW Australia; 3 Sunshine Coast Mind and Neuroscience Thompson Institute, University Sunshine Coast, Sunshine Coast, QLD Australia; 4 School of Psychological Sciences, Monash University, Clayton, VIC Australia

**Keywords:** Cannabis, Mobile health, Smartphone apps, Self-treatment, Intervention

## Abstract

**Background:**

Smartphone applications (apps) offer a promising alternative to face-to-face treatment due to their ease of access and convenience. However, there is a lack of evidence-based apps for cannabis users wishing to reduce their use.

**Objectives:**

The current study evaluated the feasibility and acceptability of a smartphone app intervention (called Assess, Plan, Track, and Tips [APTT]) for cannabis users wanting to reduce their use.

**Method:**

The current study included 111 cannabis users (68% male, aged 18–50 yrs) who had used cannabis in the past month, were not currently in treatment, and who wanted to reduce/quit their use. Participants were given access to APTT for 1 month. Participants reported on their cannabis use and related problems, confidence in resisting use, severity of dependence, and stage of change at baseline, post-intervention (4 weeks), and at 1-month follow-up. At post-intervention, participants also reported on their usage and satisfaction with the app.

**Results:**

The current study found that APTT was acceptable, with over 40% of participants using the app over 20 times over the course of a month. Participants showed a reduction in dependence and cannabis related problems over the course of the study. Further, participants’ stage of change at baseline predicted changes in cannabis use.

**Conclusions/importance:**

These findings support the feasibility and acceptability of APTT as an engaging app for cannabis users wishing to better manage their use and support the need for future RCTs to assess the efficacy of mobile-based interventions for cannabis users.

**Electronic supplementary material:**

The online version of this article (10.1186/s42238-019-0009-6) contains supplementary material, which is available to authorized users.

## Background

Cannabis is the most frequently used illicit drug in Australia. Data from the 2016 National Drug Strategy Household Survey indicates that one in three people aged 14 years and older (35%) have tried cannabis, with 10.4% using in the past 12 months. Of those who used cannabis recently, 14.4% use daily (AIHW, 2017). Approximately 10% of ‘ever users’ become dependent upon cannabis; this figure increases to 50% for daily users (Copeland and Swift 2009). Regular and/or dependent cannabis use has also been associated with cognitive impairment (Solowij and Battisti 2008), brain abnormalities (Chye et al. 2019), depressive symptoms, (Lev-Ran et al. 2014), and negative schizophrenia-like symptoms among younger users (Albertella et al. 2018).

Interventions based on cognitive behavioural therapy (CBT), motivational interviewing (MI), and personalized normative feedback (PNF) have been shown to effectively reduce cannabis use and associated problems (Copeland et al. 2001; Davis et al. 2015; Hoch et al. 2014; Martin and Copeland 2008; Riggs et al. 2018), but the majority of users do not seek professional treatment (Agosti and Levin 2004; Cunningham 2000; Stinson et al. 2006). Commonly reported barriers to seeking treatment include limited access and perceived stigma (Gates et al. 2012; van der Pol et al. 2013), with many cannabis users preferring self-reliant interventions and informal help to assist with quitting (e.g., van der Pol et al. 2013).

Technology-delivered interventions offer a promising alternative to face-to-face treatment due to their ease of access and convenience. Importantly, they have been shown to be effective in reducing cannabis use and related problems (Gates and Copeland 2017; Hoch et al. 2016; Olmos et al. 2017). For example, in an evaluation of a fully self-guided internet treatment intervention for cannabis use, participants in the active group reduced their cannabis use by 40% compared to control participants, who reduced their use by 28% (Rooke et al. 2013). In another study, participants who undertook a 50-day web-based intervention (supplemented by therapist support) showed greater reductions in their cannabis use compared to a wait-list control at the 3-month follow-up, with the between-group effect size being moderate to large (Tossman et al. 2011).

The benefits of employing technology to deliver effective cannabis use treatment interventions are perhaps best illustrated through mobile-phone technology. Most people own a mobile phone (Klasnja and Pratt 2012) and feel deeply attached to it, carrying it everywhere they go (Vincent 2006). This connection may facilitate the uptake of health interventions delivered via a mobile phone (Klasnja and Pratt 2012). There are now more than 300,000 medical or health-related applications (apps) available for download onto mobile devices (Aitken et al. 2017; Byambasuren et al. 2018). Of these, however, the number that offer evidence-based strategies to change addiction-related behaviour is considerably smaller (Tofighi et al. 2019). For instance, out of hundreds of alcohol use intervention apps on iTunes, a minority offer behaviour change techniques that are evidence or theory based (Cohn et al. 2011; Crane et al. 2015). Likewise, for smoking cessation, while a number of evidence-based mobile/app-based interventions have been developed and tested (Whittaker et al. 2016), very few are available publicly (Haskins et al. 2017). For cannabis use, there is a general lack of publicly available evidence-based apps (Ramo et al. 2015) as well as of studies examining the effectiveness of app-based interventions that are free of charge to the community.

Thus, we developed a smartphone app using cognitive-behavioural and motivation enhancement principles that have previously demonstrated efficacy in face-to-face and online treatment trials (Copeland et al. 2001; Rooke et al. 2013; Schaub et al. 2013) as well as incorporated feedback from cannabis users at various stages of development. The current study examines the feasibility and acceptability of this app, APTT (Assess, Plan, Track, & Tips), as a mobile-delivered intervention to help cannabis users wishing to reduce or quit their use. We hypothesised that APTT participants would show significant reductions in their cannabis use, cannabis problems, dependence severity, and increased confidence to resist cannabis over time. Further, as an individual’s stage of change (i.e., level of commitment to changing behaviour) has been shown to influence intervention engagement and drive behavioural changes (Connors et al. 2013), the current study will explore whether participants’ stage of change influences app engagement, perceived usefulness, and cannabis use outcomes.

## Method

### Participants

One hundred and twenty-three people completed an online screener to determine study eligibility. Inclusion criteria required that participants were at least 16 years of age or older, owned an iPhone with internet connectivity, had an email account, reported using cannabis in the previous month, had a desire to quit or reduce their cannabis use, and were fluent in English. Age, desire to reduce use or quit, and iPhone requirements were specified in the study advertisement. Those who reported acute psychiatric distress, defined using the K10 (Kessler et al. 2002) and a cut-off score of 30, or who were currently receiving treatment for cannabis use, or had done so in the previous *3* months were also excluded. Twelve people were not eligible (six due to high distress, one due to not wanting to quit/reduce, and five due to not having an iPhone). One hundred and eleven people were recruited into the study and completed the baseline assessment.

### Procedures

Ethical approval for this study was given by the University of New South Wales (UNSW Australia) Human Research Ethics Committee. Recruitment was carried out via advertisements in print and online media seeking individuals interested in reducing or quitting their use of cannabis. Upon expressing interest, individuals were sent further participant information materials and a screening assessment via email. Eligible participants were notified by email and sent a link to complete the baseline assessment. Upon completion of this assessment, participants were provided with a link to the app along with downloading instructions. Participants were asked to use the app for 4 weeks.

All assessments were conducted online. Intervention outcomes were assessed after 4 weeks’ use of APTT (post-intervention assessment) and again 1 month later (follow-up). After 4 weeks’ use of the app, access was disabled, and participants were sent an email containing a link to an online post-intervention assessment. Another email was sent 1 month later with a link to the follow-up assessment. Participants who did not complete an assessment following the initial notification received up to three reminder emails, delivered weekly, then one telephone reminder when emails were unsuccessful. Participants were reimbursed for completing each assessment ($30 voucher for the baseline assessment, and $50 voucher each for the post-intervention and follow-up assessments). At the completion of their participation in the study, participants were emailed a debriefing statement detailing the objectives of the study.

### Measures

Demographic information was collected from participants at baseline, including age, gender, and treatment history. At the post-intervention assessment, participants were asked questions relating to their usage of the app (adapted from Rizvi et al. 2011). This included “Approximately how many days did you use APTT in the past month?” (0 = I didn’t use it, 1 = 1–2 times, 2 = 3–10 times, 3 = 11–20 times, 4 = More than 20 times) and “How much time, on average, did you use APTT per day?” (0 = Less than 5 min, 1 = 5–10 min, 2 = 11–30 min, 3 = 31–60 min, 4 = More than 60 min). Participants also were asked to rate the helpfulness of APTT according to seven domains: feedback provided; setting a goal; monitoring goal progress; monitoring cannabis use; understanding reasons for use; providing strategies to manage use; motivating reduced use. Responses ranged from 1 to 5 (1 = not at all; 2 = A little; 3 = Somewhat; 4 = Very much; 5 = extremely). In addition, participants were asked to rate their satisfaction with the app using seven items from the Client Satisfaction Questionnaire (CSQ; Larsen et al. 1979), modified to include app-specific wording (e.g. “How would you rate the quality of the service you received” was modified to “How would you rate the quality of APTT”). CSQ responses were scored from 0 to 3, with total scores ranging from 0 (dissatisfied) to 21 (very satisfied).

At all three time points, days of cannabis use over the past month, severity of dependence, confidence to resist use, and cannabis-related problems were assessed. Details regarding participants’ cannabis use were collected using a modified (for online use) version of the Timeline Follow-Back method (TLFB; Norberg et al. 2012; Rueger et al. 2012; Sobell and Sobell 1996). The TLFB asked participants to estimate their cannabis use over the past 30 days using a calendar. Cannabis dependence severity was assessed using the Severity of Dependence Scale (SDS; Gossop et al. 1995), a five-item questionnaire that produces a total score from 0 to 15, with higher scores indicating more severe dependence symptoms. The Cannabis Problems Questionnaire (CPQ; Copeland et al. 2005) consists of 20 yes/no response items to produce a total score out of 20 (higher scores indicate more problems) and has demonstrated good psychometric properties. Other outcomes of interest included confidence to resist using cannabis, which was measured using the eight-item Drug Taking Confidence Questionnaire (DTCQ-8) where participants rated on a scale of 0 to 100% their confidence to resist cannabis in 8 different situations (Sklar and Turner 1999).

Finally, participants’ stage of change was measured using the Readiness to Change Questionnaire (RCQ; Heather et al. 1991), a 12-item questionnaire based on the stages of change model (Prochaska and DiClemente 1982). The RCQ was scored using the quick method, which allocates an individual according to one of three stages of behavior change (Precontemplation, Contemplation, and Action) based on the highest scale score. Where there are ties between stage scores, allocation is made to the higher motivational stage. Notably, all study participants fell into either the Contemplation or Action stages, with the exception of three participants in the Precontemplation stage. These participants were thus allocated to the Contemplation stage.^1^

### Intervention components

APTT comprised four modules: Assess, Plan, Track, and Tips. The Assess module assessed current levels of cannabis use, reasons for use, and perceived consequences. Personalised and normative feedback on cannabis use and cannabis-related problems was then provided, which could be saved for later viewing as well as forwarded to a nominated email address. This feedback report included information comparing the participant’s cannabis use to the general Australian population (age and gender matched)^2^; how much money they would save a week/year/twenty years if they stopped smoking; the number of cannabis abuse and dependence symptoms endorsed; self-reported pros and cons of cannabis use, reasons for cannabis use, and the negative consequences endorsed. After reading through the feedback, users were prompted to create a plan for reducing or quitting cannabis, which they could do so immediately or at a later stage.

The Plan module assisted users to choose a goal and create a plan to quit or reduce their cannabis use. Participants who used cannabis daily and opted to quit were provided with additional information on managing withdrawal and offered a reduction schedule (i.e., reduce daily use by one-third each day for 7 days) to minimise withdrawal (See Additional file 1). Participants did not have to accept the reduction schedule. Strategies to achieve goals were then provided based on participants’ chosen reasons for use (e.g. to be liked/not feel left out; to feel good/get high; to relax/sleep/forget problems; to boost awareness/creativity; to be sociable/more confident). Participants could select and save their preferred strategies as ‘favourites’. All reason-specific and general strategies were accessible in the Tips module. See Additional file 1 for a range of screenshots demonstrating the format of the Tips module, as well as examples of strategies used.

To monitor progress towards goals, the Track module was designed for users to record daily their cannabis use (including if they had not used), the money they spent on cannabis, and their reasons for use. To encourage users to track their use, a daily prompt was provided, which could be switched off for those who preferred no reminders. Tracking information could be viewed in graphs and infographic formats and participants received a certificate of achievement (optional, via email) when they reached their goal.

The Tips module contained a comprehensive list of strategies to help users cope when faced with a range of triggers or situations that might lead them to using cannabis. Participants could refer to these strategies at any time and could select or deselect their preferred strategies for prominent display in the app.

**Fig. 1:**
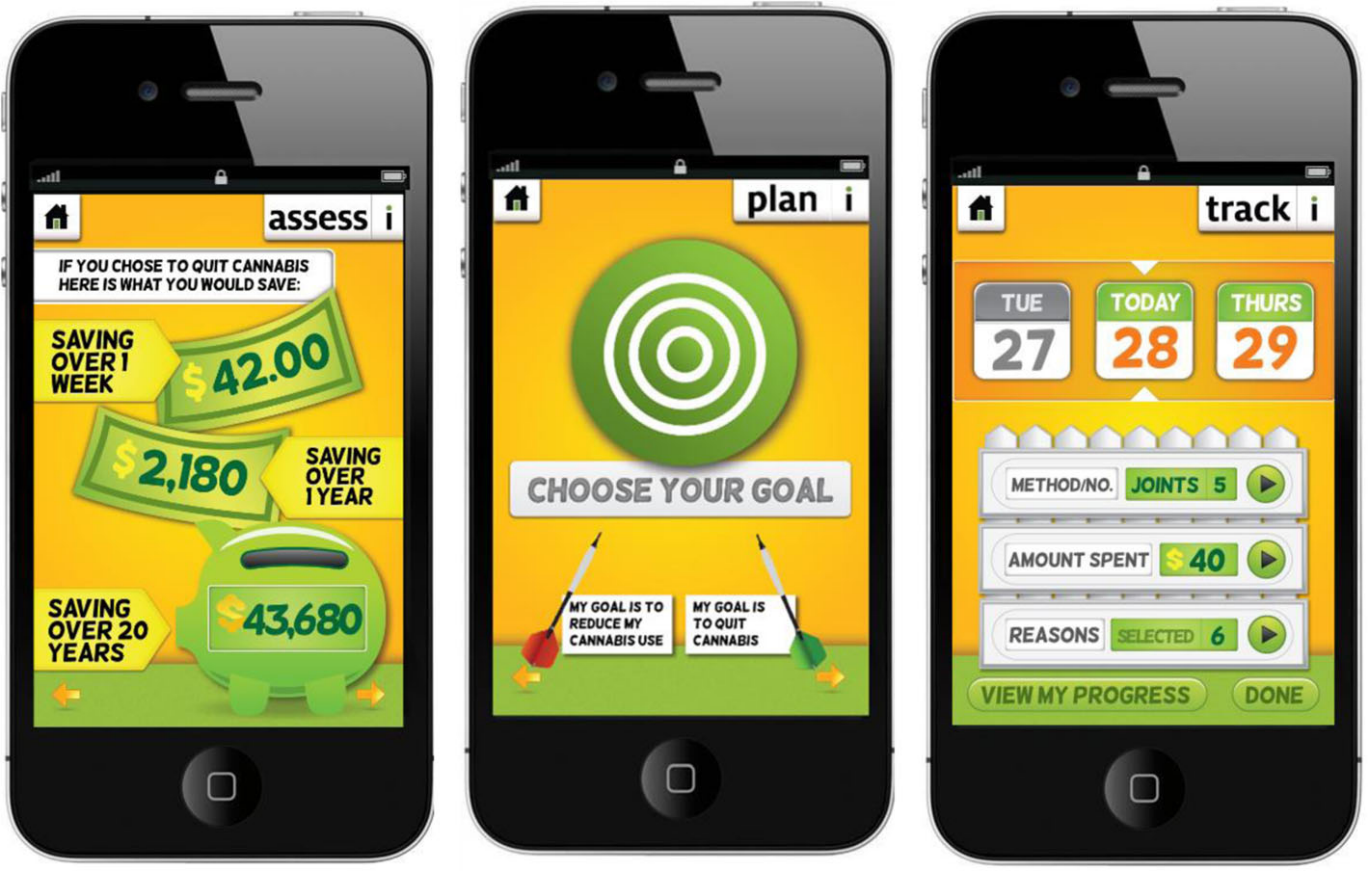
Three exemplar screenshots of APTT

In addition to these four functions, APTT was password protected and allowed users to email themselves personalised APTT content (e.g., feedback report, plan details, etc.). Lastly, users could choose either a male or female avatar or no avatar to guide them through the modules. Example screenshots are shown in Fig. 1.

### Data analysis

The data were analysed using Generalised Estimating Equations (GEE), allowing for all participants to be entered into the analysis, even with missing data at one or both of the follow-up points. Supplementary Intention To Treat (ITT) analyses were also carried out, which used a last case carried forward (LCCF) approach, to examine the influence of attrition on study findings. These ITT analyses are provided in the Additional file 1. For all analyses, an auto-regressive (1) correlation matrix was used. The cannabis use outcomes that were analysed as dependent variables included: cannabis use (number of days), cannabis problems (CPQ), cannabis dependence (SDS), and confidence to resist use (DTCQ-8). Days were analysed using a Poisson model with log link function. CPQ data had a normal distribution when considered across assessment time points, and was analysed using a linear model. DTCQ data were not normal and accepted techniques designed to transform it for further analyses failed. Thus, we dichotomized DTCQ scores according to confidence status (not confident in resisting - less than 50% versus confident in resisting - 50% and over), and analysed it using a binary logistic model. SDS data had a negative binomial distribution and thus was analysed using a negative binomial model. Time and Stage of Change (RCQ: Contemplation versus Action) were entered as factors and their interaction assessed. Covariates included gender, past quit attempt/s, and goal type (Reduce versus Quit), as these have been shown to influence motivation to change and/or outcomes in addiction-related interventions (Biener and Abrams 1991; Peters et al. 2007; Thrul et al. 2014; Ward et al. 1997) Corrections were applied for number of tests (.05/4 = .015). Significant interactions (between RCQ status and Time were followed up by comparing RCQ groups at post-intervention and follow-up. Follow-up group comparisons and participant usage and satisfaction data were analysed using Mann-Whitney U tests.

## Results

Of the 111 participants who completed the baseline assessment, 93 completed the post-intervention assessment (16% attrition), and 75 completed the one-month follow-up assessment (32% attrition). Attrition analyses were carried out to compare participants who completed all three assessments against those who dropped out either at the post or one-month follow-up on age, gender, RCQ status, and baseline scores on each of the four outcome variables. Between. These are presented in Table 1; notably, no significant differences were found.

**Table 1:** Descriptive statistics comparing participants who completed all three assessments (Completers) versus those who were lost to follow-up (Non-completers)

		Completers (*n* = 75)	Non-completers (*n* = 36)	*p*
Gender^c^	Female %	32%	44%	.201
Age^t^	Mean	27.0	26.2	.626
	SD	7.60	8.37	
Days^m^	Md	28.0	30.0	.056
	min - max	4–30	7–30	
SDS^a,^^m^	Md	6.0	5.5	.695
	min - max	0–13	0–12	
DTCQ^c^	Confident %	37%	28%	.321
CPQ^t^	Mean	7.1	7.3	.689
	SD	3.46	3.41	
Reduce vs Quit^c^	Quit %	32%	28%	.651
Past quit attempt/s^c^	Yes %	76%	75%	.908
RCQ^c^	Action %	35%	33%	.890

^a^Completers, *n* = 66; Non-completers, *n* = 32

Note: ^c^ indicates Chi-square test; ^t^ indicates t-test; ^m^ indicates = Mann-Whitney U test

Participants were 111 cannabis users, primarily male (64%) and aged between 18 and 50 years of age (mean = 26.7, SD = 7.8). The majority (79%) were born in Australia, working full-time (46%), and had obtained a diploma or trade-level certificate (42%). Seventy-five percent of participants were classified as dependent using the cut-off of 3 and above for cannabis dependence according to the SDS (Swift et al. 1998). Three quarters (76%) of participants reported at baseline that they had previously made an attempt to quit their cannabis use, though just 11% had sought professional help for their cannabis use in the past. Sixty-nine percent of participants signed up to the app with the goal of reducing their cannabis use, and the remaining 31% wanted to quit. Participants were divided into either the contemplation stage (66%) or action stage (34%) of readiness to change. There was no association between stage of change and goal, *p* > .10.

Self-reported usage data and participant satisfaction data was collected at the post-intervention assessment and is reported in Table 2. Over 40% of participants reported using the app over 20 times in the past month. Only two participants (2%) did not use the app at all. Participants in the Action Stage found APTT more motivating in terms of helping them manage their cannabis use, *Z* = − 2.14, *p* = .033. There was no group difference between participants in the Contemplation stage (*Md* = 11, 1–18, *n* = 59) and those in the Action stage (*Md* = 12, 4–18, *n* = 34), *Z* = − 1.64, *p* = .102 on CSQ score.

**Table 2:** Comparison of participants in the Contemplation stage (*n* = 59) against participants in the Action stage (*n* = 34) in relation to app usage and satisfaction

How helpful was APTT in…		Not at all	A little	Somewhat	Very much	Extremely	Md (min - max)	*p*
Providing feedback?	Cont.	12%	20%	31%	27%	10%	2 (0–4)	.559
	Action	3%	24%	29%	41%	3%	2 (0–4)	
Setting a goal?	Cont.	12%	31%	34%	17%	7%	2 (0–4)	.228
	Action	6%	32%	21%	32%	9%	2 (0–4)	
Monitoring goal progress?	Cont.	9%	24%	22%	41%	5%	2 (0–4)	.542
	Action	6%	27%	21%	29%	18%	2 (0–4)	
Monitoring cannabis use?	Cont.	9%	12%	20%	42%	17%	3 (0–4)	.238
	Action	0%	21%	15%	32%	32%	3 (1–4)	
Understanding reasons for use?	Cont.	29%	29%	14%	24%	5%	1 (0–4)	.085
	Action	9%	32%	27%	24%	9%	2 (0–4)	
Providing strategies to manage use?	Cont.	32%	29%	20%	14%	5%	1 (0–4)	.338
	Action	18%	35%	29%	18%	0%	1 (0–3)	
Motivating reduced use?	Cont.	25%	27%	32%	12%	3%	1 (0–4)	.033*
	Action	6%	35%	27%	21%	12%	2 (0–4)	
		Less than 5 mins (0)	5–10 min (1)	11–30 min (2)	31–60 min (3)	More than 60 mins (4)	Md (min – max)	
How much time on average did you…	Cont.	66%	25%	7%	2%	0%	0 (0–3)	.303
	Action	53%	41%	6%	0%	0%	0 (0–2)	
		I didn’t use it (0)	1–2 times (1)	3–10 times (2)	11–20 times (3)	More than 20 times (4)		
How many times did you use APTT in the past month	Cont.	0%	3%	20%	29%	48%	3 (1–4)	.058
	Action	6%	0%	38%	24%	32%	3 (0–4)	

* *p* < .05

**Fig. 2:**
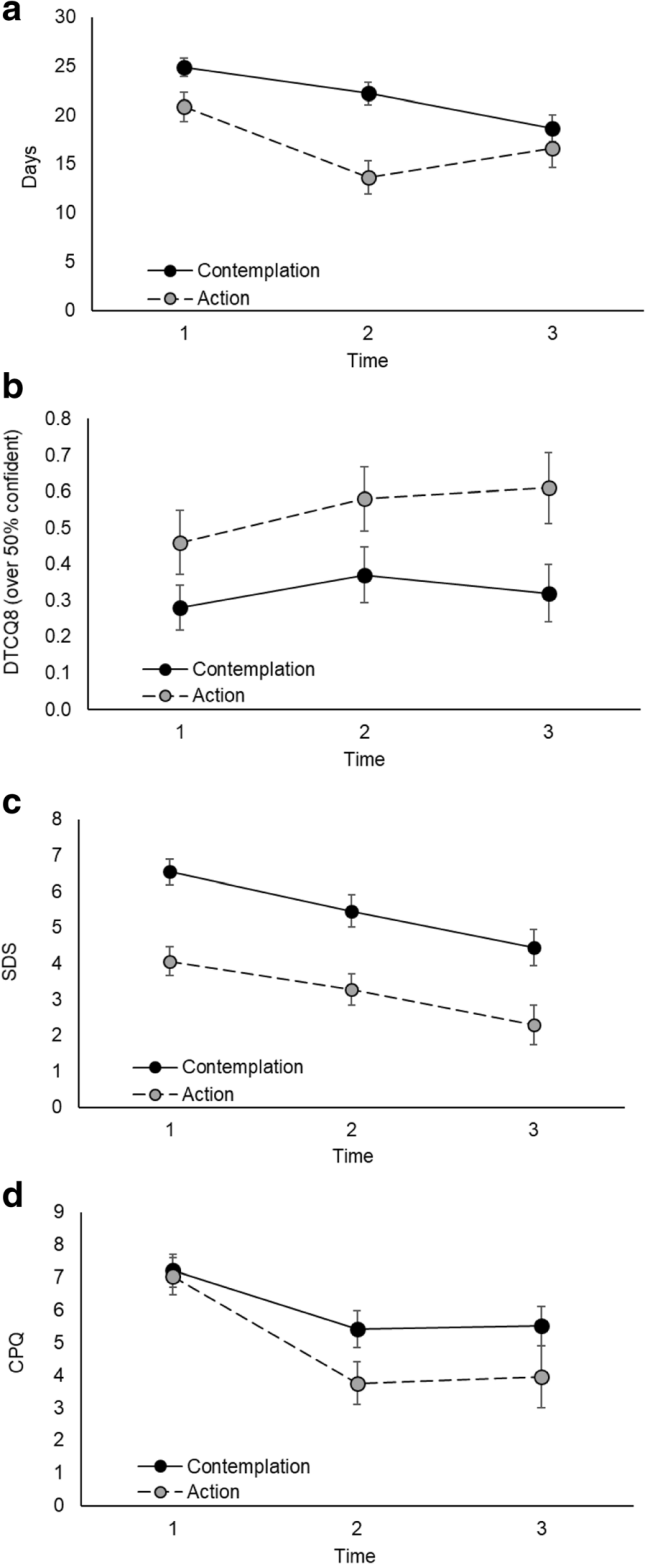
Estimated marginal means for participants in the Contemplation (black circles, full line) and Action (grey circles, dashed line) stages of change from corresponding GEE regressions on: **a** Cannabis use (number of days), **b** Confidence to resist using cannabis (DTCQ-8), **c** Severity of dependence (SDS scores), and **d** Cannabis problems (CPQ scores)

Table 3 contains descriptive statistics of the cannabis use variables analysed at baseline, post-intervention, and follow-up, for those participants who completed the corresponding assessment. The results of the GEEs for each outcome are shown in Table 4. The GEE on Days used in the past month found a significant interaction between Time and RCQ stage, Wald *χ*^2^ = 11.59, *p* = .003, which parameter estimates (not shown in Table 3) suggest was driven by differences between RCQ status groups at post-intervention, Wald *χ*^2^ = 6.35, *p* = .012. A follow-up Mann-Whitney U test found that the Action group (Md = 24.5, 0–30) used cannabis less days than the Contemplation group (Md = 12.0, 0–30) at post-intervention, *Z* = − 3.06, *p* = .002. This difference was no longer seen at follow-up, *Z* = − 1.16, *p* = .248. Figure 2a, which shows number of days (estimated marginal means) as a function of RCQ status over time. The GEE on DTCQ found a significant association between RCQ status and confidence to resist such that those in the Action stage had higher levels of confidence overall, Wald *χ*^2^ = 8.54, *p* = .003. Figure 2b shows confidence (estimated marginal means) (%) as a function of RCQ status and time. The GEE on SDS found a significant association between RCQ status and dependence scores, Wald *χ*^2^ = 16.03, *p* < .001, with participants in the Action stage having lower dependence overall. Further, participants wanting to quit (versus reduce) showed greater dependence overall, Wald χ^2^ = 17.43, *p* < .001. There was also a significant effect of time, Wald *χ*^2^ = 13.92, *p* = .001. Figure 2c shows the estimated marginal means of SDS as a function of time and RCQ status. Finally, the GEE on CPQ found a significant effect of time. The estimated marginal means corresponding to this analysis are shown in Fig. 2d.

**Table 3:** Cannabis use measures across study assessment points

		Baseline (111)	Post (93)	Follow-up (75)
Days	Md	29	21	20
	Range	4–30	0–30	0–30
DTCQ (%)	Md	40%	50%	48%
	Range	0–100%	0–93%	0–100%
CPQ	M	7.1	4.8	5.0
	sem	.33	.39	.50
SDS^a^	Md	6	4	3
	Range	0–13	0–14	0–14

*SDS* Severity of Dependence, *CPQ* Cannabis Problems Questionnaire, *DTCQ* Drug Taking Confidence Questionnaire, Days (cannabis use days in past month)

^a^ Ns = 98/93/75

**Table 4:** GEE regression results

	B	SE	Exp(B)	Wald X^2^	*p*
Dependent variable: Days used (*n* = 111/93/75)					
Time	–	–	–	27.512	< .001
RCQ	–	–	–	8.411	.004
RCQ x Time	–	–	–	11.593	.003*
Gender	.064	.0652	1.066	.959	.327
Reduce vs Quit	.117	.0592	1.124	3.887	.049
Past attempt	.145	.0906	1.156	2.550	.110
Dependent variable: DTCQ8 (*n* = 111/93/75)					
Time	–	–	–	3.274	.195
RCQ	–	–	–	8.543	.003*
RCQ x Time	–	–	–	.780	.677
Gender	.115	.3397	.892	.114	.735
Reduce vs Quit	−.433	.3501	1.541	1.528	.216
Past attempt	−.392	.3475	1.480	1.272	.259
Dependent variable: SDS score (*n* = 98^a^/93/75)					
Time	–	–	–	13.920	.001*
RCQ	–	–	–	16.034	< .001*
RCQ x Time	–	–	–	.466	.792
Gender	−.038	.1246	.962	.095	.758
Reduce vs Quit	.458	.1097	1.581	17.430	< .001*
Past attempt	.302	.1293	1.353	5.465	.019
Dependent variable: CPQ score (*n* = 111/93/75)					
Time	–	–	–	61.172	< .001*
RCQ	–	–	–	2.824	.093
RCQ x Time	–	–	–	5.278	.071
Gender	−.082	.6818	.921	.014	.904
Reduce vs Quit	1.159	.7092	3.185	2.669	.102
Past attempt	1.445	.7204	4.240	4.020	.045

^a^some participants had items of the SDS questionnaire missing at baseline due to a questionnaire error. These participants were omitted from these analyses

* *p* < .015

The results from the ITT analyses revealed similar results across all outcomes (see Additional file 1).

## Discussion

The present study aimed to explore the feasibility and acceptability of a newly developed smartphone application, APTT, designed to assist cannabis users to reduce or quit their use of the drug. To the authors’ knowledge this is one of the first apps for the self-management of cannabis use to be trialled, which is grounded in evidence-based intervention principles. The findings of the current study suggest that APTT is a feasible and acceptable mobile-based intervention for cannabis users wishing to reduce or quit their use.

Stage of change predicted app perceptions; Participants in the Action stage rated the app as more motivating than participants in the Contemplation stage. Interestingly, there was a trend toward participants in the Action stage using the app less frequently than Contemplators. This latter finding might reflect the different purposes for which the app was being used. For instance, Contemplators might be using the app primarily for tracking their use, while participants in the Action stage more for accessing strategies and motivational support. This highlights the importance of measuring engagement in various ways, as successful engagement might not necessarily be a matter of quantity. Indeed, this may explain mixed findings in past research; while readiness for change should theoretically predict client engagement in a program, many studies have not found this (Choi et al. 2015; Sloas et al. 2017). Future research asking treatment users themselves what they consider successful engagement to look like might be fruitful. In relation to mobile app engagement specifically, future research examining which domains of mobile app user engagement best predict outcomes is needed.

The current study found significant reductions over the course of the study for cannabis use, cannabis-related problems, and severity of dependence. This finding should be considered with some caution as no comparison group was included in this pilot study, and it is common for even control group participants to show improvements on outcome measures, particularly among treatment seekers, as was the case for the participants in this study. However, in other online treatment studies, waitlist control participants have been shown to reduce their cannabis use by around 17% (3-month follow-up) (e.g., Tossmann et al. 2011). In the current study, cannabis use days reduced by 20% (from baseline to post-intervention). Notably, unlike other studies, there was no minimum use threshold for inclusion into the study (other than having used cannabis in the past month and wanting to reduce or quit use). Looking at just participants in the Action stage (who might be argued to be more in line with treatment-seeking samples in other studies), cannabis use reduced on average by 29% (in contrast to 14% in Contemplation stage). Such reductions, in the absence of a minimum use threshold for entry, and from such an easily accessible and low-cost intervention warrant attention for further study in an RCT.

Participants in the Action stage differed from participants in the Contemplation group in terms of changes in cannabis use days over the course of the study. At post-intervention, participants in the Action group used cannabis less days than the Contemplation group. As shown in Fig. 2a, this difference was no longer present at follow-up.

The study has some limitations that are worthy of consideration. First, this was a non-controlled study to examine whether participants would use APTT and to gauge the feasibility of doing a larger trial Thus, it cannot be known whether the changes seen in cannabis use/problems were a result of the intervention itself or some other unrelated factor. Second, we were unable to monitor participants’ actual use of the app, including the use of different functions and time spent using it, due to the cost of designing an app with that capability. The method used in the current study to assess engagement was limited in various ways, such as app usage items not being specific to features of the app (e.g., times used monitoring functions), which would have been useful in supporting our interpretation that people in the Contemplation stage used the app more frequently because they were using it to track their use. Also, self-reported usage data is subject to bias and memory influences. Recently, alternative methods of gauging usage data have emerged, including freely downloadable apps designed specifically to collect this information about other apps. Such app usage information will provide a more objective measure of engagement and should be used in future studies. Finally, we did not examine whether using the app motivated continued help-seeking for those that did not meet their initial goal of reducing/quitting.

Future directions, aside from addressing the above noted limitations, include a version of APTT modified for use in conjunction with face-to-face treatment. Additional modifications/extensions include a cognitive training module, which could enhance the learning and implementation of strategies through the improvement of cognition (Bickel et al. 2014), and a harm reduction module, which could cover topics such as using high CBD strains (which may be protective against harms associated with THC, Niesink and van Laar 2013), vaping instead of smoking, and information of interactions of cannabis with other drugs where evidence is available to support their inclusion in a clinical intervention.

## Conclusion

Advancement in technology has brought new ways in which a wide range of health interventions can be developed and delivered. Current interventions for cannabis users are limited by low uptake due to accessibility and stigma concerns. APTT offers the advantage of convenient delivery via a smartphone, minimising considerably concerns about accessibility and stigma. The current study offers strong support for APTT’s feasibility and acceptability as an intervention for cannabis users wishing to manage their use.

## Additional file

Additional file 1: Supplementary materials to accompany 'A Smartphone App Intervention for Adult Cannabis Users Wanting to Quit or Reduce Their Use: A Pilot Evaluation'. (DOCX 2601 kb)

## Data Availability

Available upon request.

## Abbreviations

APTT: Assess, Plan, Tips, and Track
CPQ: Cannabis Problems Questionnaire
CSQ/CSQ-7: Client Satisfaction Questionnaire (7 items)
DTCQ/DTCQ-8: Drug Taking Confidence Questionnaire (8 items)
GEE: Generalised Estimating Equations
ITT: Intention to Treat
LCCF: Last case carried forward
RCQ: Readiness to Change Questionnaire
SDS: Severity of Dependence Scale
TLFB: Time Line Follow Back

## Footnotes

1 Re-running the analyses with these participants excluded did not change the results.

2 Data (from 2007 to 2010) obtained directly from the Australian Government Department of Health
