# Correction to: A smartphone app intervention for adult cannabis users wanting to quit or reduce their use: a pilot evaluation

**DOI:** 10.1186/s42238-019-0010-0

**Published:** 2019-11-01

**Authors:** Lucy Albertella, Lisa Gibson, Sally Rooke, Melissa M. Norberg, Jan Copeland

**Affiliations:** 1 National Cannabis Prevention and Information Centre, UNSW Sydney, Kensington, NSW Australia; 2 Centre for Emotional Health, Department of Psychology, Macquarie University, North Ryde, NSW Australia; 3 Sunshine Coast Mind and Neuroscience Thompson Institute, University Sunshine Coast, Sunshine Coast, QLD Australia; 4 School of Psychological Sciences, Monash University, Clayton, VIC Australia

**Correction to: J Cannabis Res (2019) 1:9**

**https://doi.org/10.1186/s42238-019-0009-6**

Following publication of the original article (Albertella et al. 2019), the authors have flagged an error concerning the reference to the ‘follow-up Mann-Whitney U test’ in the Results section of the article.

The error is that the median values in brackets should be switched: the Action group should have the lower median value of 12.0, while the Contemplation group should have the higher value of 24.5.

NB the corresponding information in Fig. 2a is correct; the error affects just the above mentioned median values.

The authors apologize for this error.
