# Supplementary material for: A smartphone app intervention for adult cannabis users wanting to quit or reduce their use: a pilot evaluation
Source: J Cannabis Res. 2019 Aug 16;1:9. doi: 10.1186/s42238-019-0009-6 (PMC7819295; doi:10.1186/s42238-019-0009-6)

SUPPLEMENTARY MATERIALS

TO ACCOMPANY:

**A Smartphone App Intervention for Adult Cannabis Users Wanting to Quit or Reduce Their Use: A Pilot Evaluation**

**Table S1. Results from the GEE analyses using ITT (last case carried forward) approach.**

Days – Poisson GEE (n=111)

|  | B | SE | Wald Chi-Square | p |
| --- | --- | --- | --- | --- |
| Time |  |  | 9.797 | .002 |
| RCQ |  |  | 24.989 | .000 |
| RCQ x Time |  |  | **11.255** | **.004** |
| Gender | .069 | .0679 | 1.020 | .313 |
| Reduce vs Quit | .145 | .0911 | 2.515 | .113 |
| Past attempt | .103 | .0613 | 2.818 | .093 |

DTCQ - Binary logistic GEE (n=111)

|  | B | SE | Wald Chi-Square | p |
| --- | --- | --- | --- | --- |
| RCQ |  |  | **7.089** | **.008** |
| Time |  |  | 1.847 | .397 |
| RCQ x Time |  |  | .311 | .856 |
| Gender | .184 | .3396 | .292 | .589 |
| Past attempt | -.419 | .3645 | 1.323 | .250 |
| Reduce vs Quit | -.440 | .3508 | 1.575 | .210 |

SDS – Negative Binomial GEE (n = 98)

|  | B | SE | Wald Chi-Square | p |
| --- | --- | --- | --- | --- |
| RCQ |  |  | **18.076** | **<.001** |
| Time |  |  | **18.168** | **<.001** |
| RCQ x Time |  |  | .753 | .686 |
| Gender | -.022 | .1299 | .027 | .868 |
| Past attempt | **.285** | **.1317** | **4.699** | **.030** |
| Reduce vs Quit | **.441** | **.1145** | **14.853** | **<.001** |

CPQ - Linear GEE (n=111)

|  | B | SE | Wald Chi-Square | p |
| --- | --- | --- | --- | --- |
| RCQ | - | - | 3.130 | .077 |
| Time | **-** | **-** | 54.088 | <.001 |
| RCQ x Time | **-** | **-** | **6.302** | **.043** |
| Gender | -.170 | .6772 | .063 | .801 |
| Past attempt | 1.457 | .7552 | 3.721 | .054 |
| Reduce vs Quit | 1.095 | .7115 | 2.368 | .124 |

Bolded if p < .0125 (i.e., .05/4)

**Table S2. Strategies by reason for use**

| *To be liked/not feel left out* |
| --- |
| - Before going out, I will decide how many joints/cones I will allow myself to have |
| - I will let my friends know that I am trying to reduce/quit cannabis and ask them to support my decision |
| - If offered a smoke, I will simply say ‘no thanks, I don’t smoke anymore’ |
| - I will identify friends who don’t use cannabis or who are also trying to reduce cannabis or quit and I will stick with them in social situations |
| - I will suggest activities that do not involve cannabis |
| - I will leave when people are using cannabis |
| - I will use strategies to fit in, such as listening and showing interest in what other people have to say |
| - I will avoid social situations where people are likely to be smoking |
| - I will engage in new social activities with people who do not smoke cannabis |
| - I will seek help for improving my social skills (e.g. toastmasters, self-help books) |
|  |
| *To feel good/get high* |
| - I will find other ways to have a good time (e.g. rock climbing, going to the movies, camping etc.) |
| - I will think about how good I will feel tomorrow knowing I am one step closer to reaching my goal |
| - I will tolerate not being high and deal with it by allowing myself time to learn that I can have a good time without cannabis |
| - I will fill up my spare time with enjoyable non-smoking activities |
| - I will take up a new hobby |
| - I will exercise to increase my levels of endorphins (the ‘feel-good’ chemicals) |
| - I will appreciate pleasant non-cannabis using experiences |
| - I will remind myself that over time not using will become less boring |
| - I will accept that life has ups and downs and that I don’t need to be up all the time |
| - I will think back to times I had fun without cannabis and find ways to increase those activities |
|  |
| *To relax/sleep/forget problems* |
| - I will remember that using cannabis only helps me feel better in the short-term, but makes me feel worse in the long-term |
| - I will challenge my interpretation of what is upsetting me by asking: what is the evidence? Am I imaging the worst? Is there a more realistic way of interpreting the situation? |
| - I will problem-solve: I will define the problem, list all possible solutions, decide on a course of action, and carry it out |
| - I will seek professional help for concerns that contribute to my cannabis use |
| - I will use healthy coping strategies (e.g. go for a walk, call a friend etc.) |
| - I will visit the mindhealth connect website [http://www.mindhealthconnect.org.au/] for advice on coping with depression and anxiety |
| - I will practice deep breathing and relaxation exercises |
| - I will practice good sleep habits such as: getting up at the same time every day regardless of how much I slept, not napping, avoiding caffeine after midday, going to bed only when I am sleepy, avoiding light, especially from computers, tablets and phones during sleeping hours |
| - I will remind myself that change might not happen overnight but that if I stick to good sleeping habits, my sleep will improve |
| - I will visit the sleep health foundation’s website [<http://www.sleephealthfoundation.org.au/>] for advice on good sleep habits and dealing with sleep problems |
| - I will keep a notepad by my bed to write down worries or reminders of things to address the next day |
| - I will exercise regularly as exercise increases levels of endorphins and serotonin, which can lift my mood, regulate sleep, and reduce pain |
| - I will visit the Australian pain management association’s website [<http://www.painmanagement.org.au/>] for advice on dealing with pain |
| - I will use guided imagery to relax: I will close my eyes, imaging being somewhere enjoyable, and use all my senses to be fully immersed in the experience |
| - I will think of a time in my life when I overcame a major problem/challenge and remind myself of the strength I have within me |
|  |
| *To boost awareness/creativity* |
| - I will remind myself that repeated cannabis use will limit my ability to think creatively |
| - I will remind myself that true creativity does not depend on cannabis |
| - I will practice being creative without using cannabis |
| - I will remind myself of the times I have achieved creativity without using cannabis |
| - I will find other ways to enhance my creativity, like using my non-dominant had to increase communication in different areas of my brain |
| - I will avoid creative activities in which I use cannabis until I am confident that I can do them without cannabis |
| - I will engage in creative activities in which I used cannabis only when I am with non-smokers |
| - When engaging in creative activities. I will ensure that cannabis is not available to me |
| - When encountering a problem, I will think of a least 5 solutions before acting on one, which will foster creativity |
| - If I am stuck for ideas, I will open a book to a random page, pick a random word, and come up with ideas using this word |
|  |
| *To be sociable/more confident* |
| - Before going out, I will decide how many joints/cones I will allow myself to have and stick to it |
| - I will schedule something important after the social event to encourage myself not to use |
| - I will engage in social activities where cannabis use is not OK (e.g. visiting family, playing a sport etc.) |
| - I will identify friends who don’t use cannabis and stick with them in social situations |
| - I will keep myself engaged at events by listening and showing interest in what other people are saying |
| - I will remind myself that, with time and practice, I will enjoy social events even more without using cannabis |
| - I will remind myself of the times my cannabis use has ruined a social event |
| - I will accept that the type of fun I will have without cannabis will be different |
| - I will remind myself that chronic cannabis use leads to decreased sociability |
| - I will avoid cannabis using situations |
|  |
| *General* |
| - I will focus on the negative consequences of my cannabis use |
| - I will focus on the reasons why I want to reduce/quit using cannabis |
| - I will tell others about my goal and ask them to support me in achieving it |
| - I will get rid of all cannabis and cannabis-related objects in my home |
| - I will distract myself and do something else (like go for a walk, call a friend, etc.) |
| - I will delay my decision to smoke by 30 minutes |
| - I will think about the money I can save as a result of reducing/quitting cannabis |
| - I will remind myself that I can choose to overcome using cannabis |
| - I will fill up my spare time in advance with enjoyable non-smoking activities |
| - I will avoid situations and places associated with cannabis |
| - I will visualise being a non-cannabis user and start to be that person |
| - I will remind myself that cravings are time-limited to less than 30 minutes |
| - I will focus on knowing that making it through one craving will make dealing with future cravings easier |
| - I will remind myself that although cravings are unpleasant, they are not intolerable |
| - I will develop a plan to manage the short-term (1-2 weeks) distress associated with withdrawal |
| - I will remind myself that withdrawal symptoms are positive signs that my body is adjusting to life without cannabis |
| - I will remember a time when I was successful in not using cannabis and use what I did then to help me succeed |
| - I will reward myself when I reach my reduction goal |
| - I will think about how my cannabis use is hurting the people I care about |
|  |

**Figure S1. Additional example screenshots from APTT**

a) Assess module


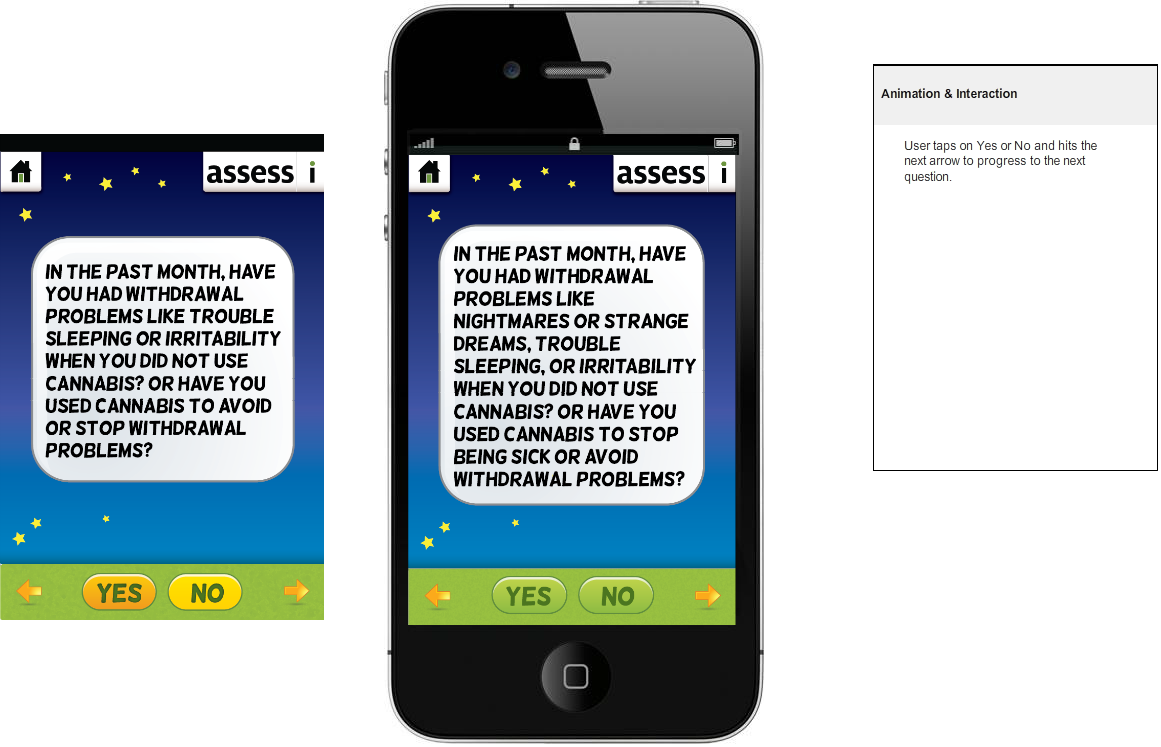

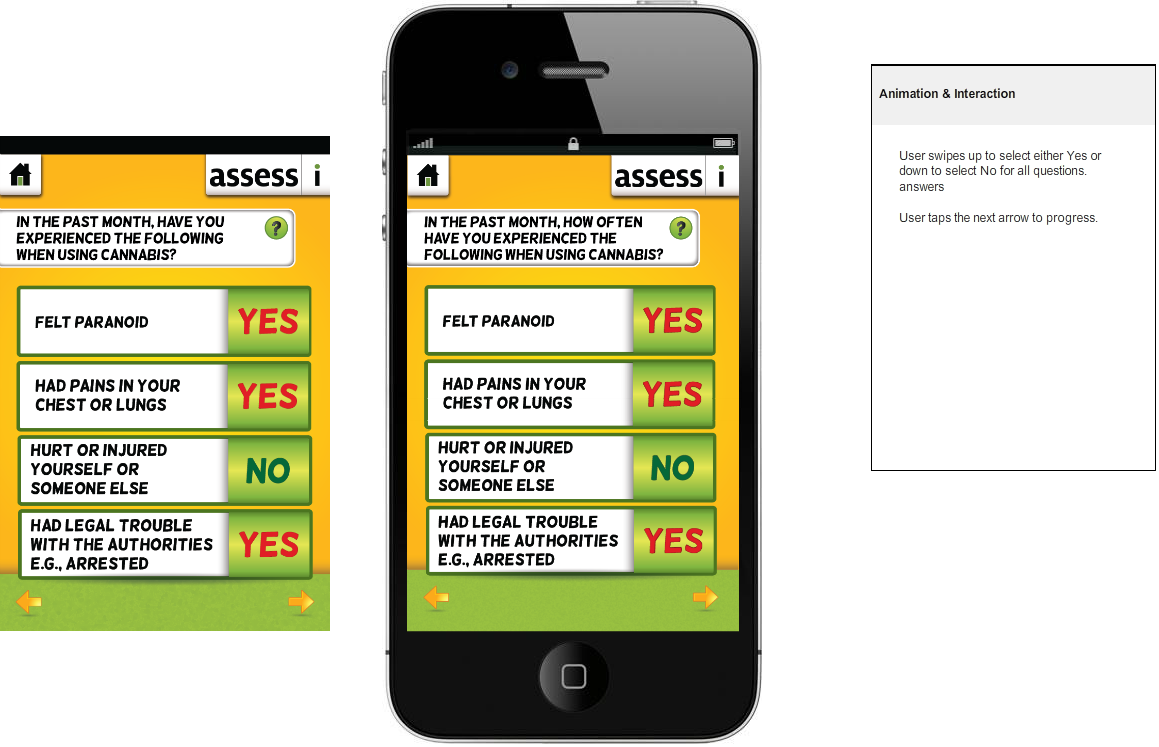

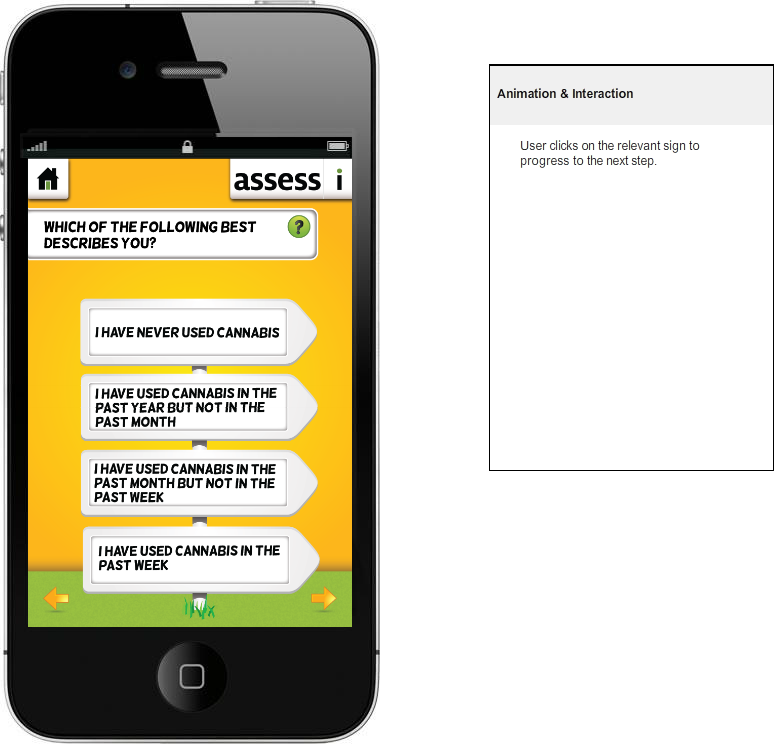


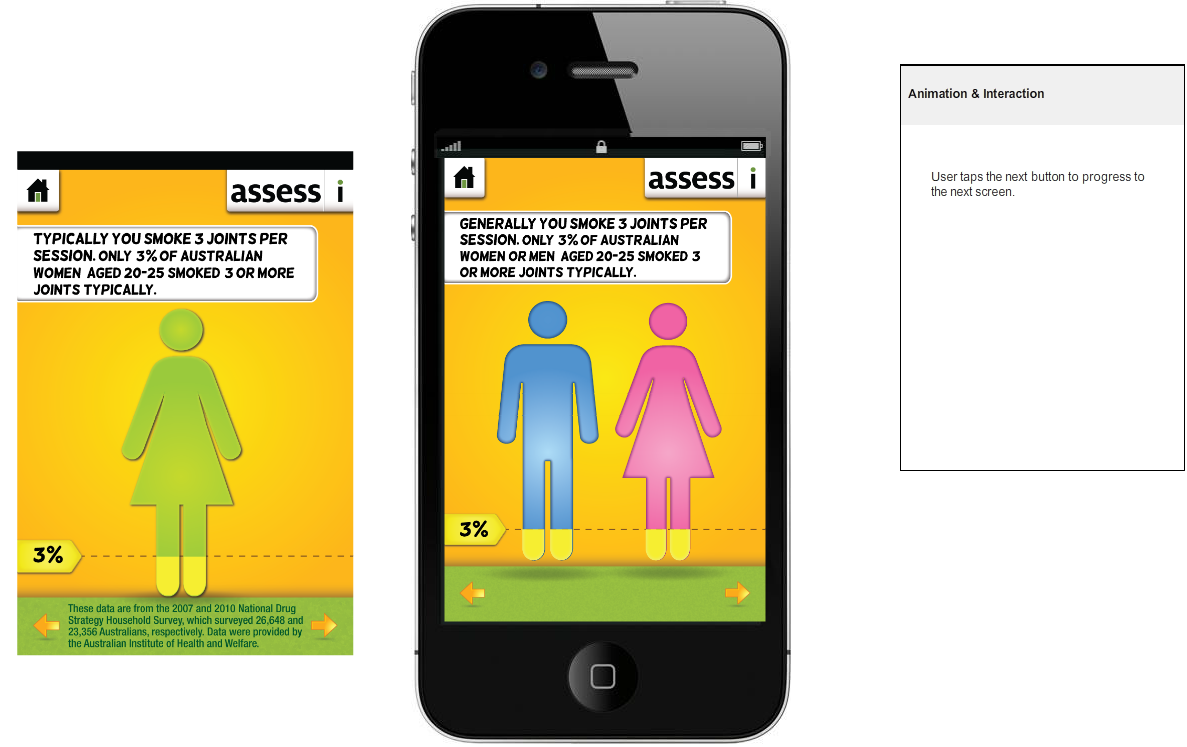

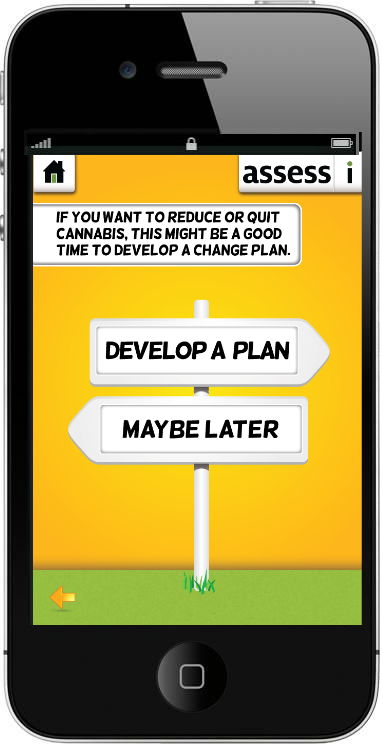

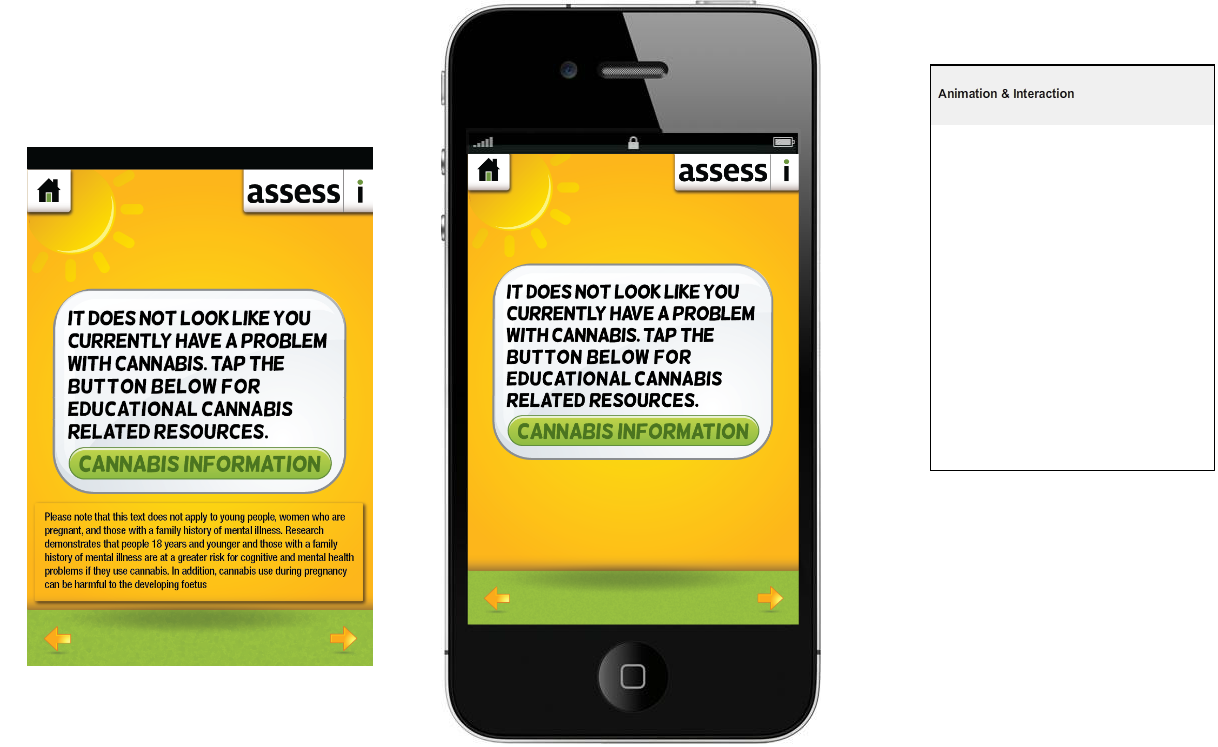


b) Plan module


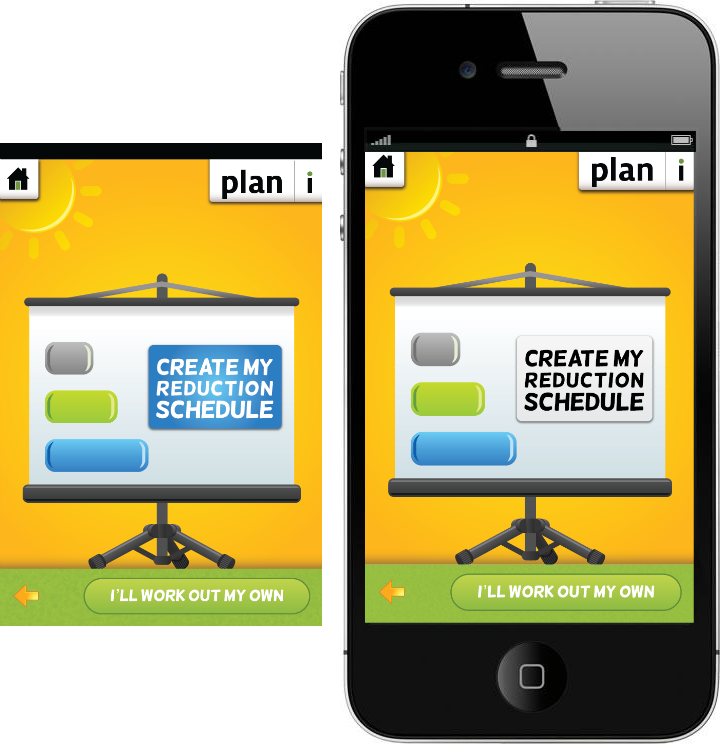

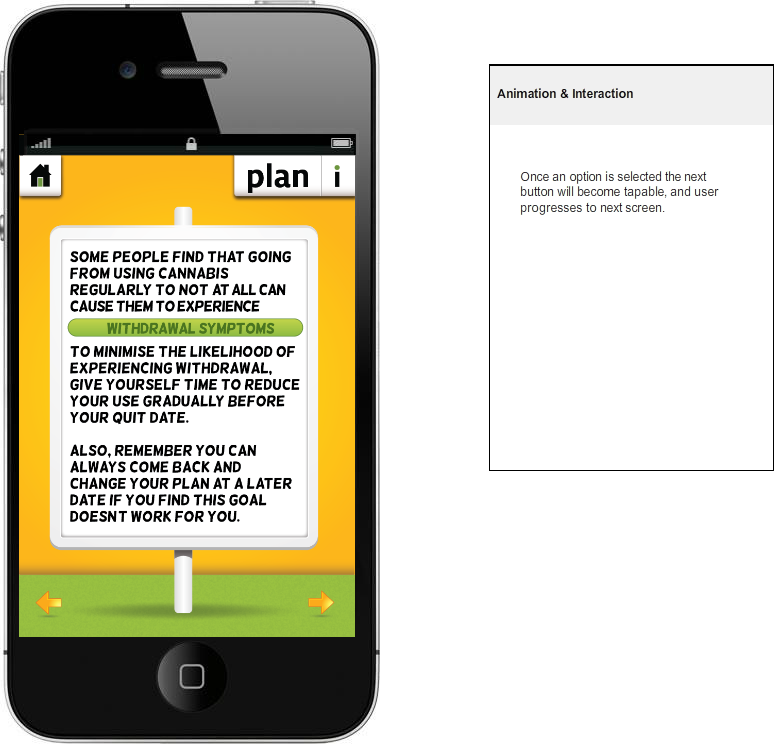

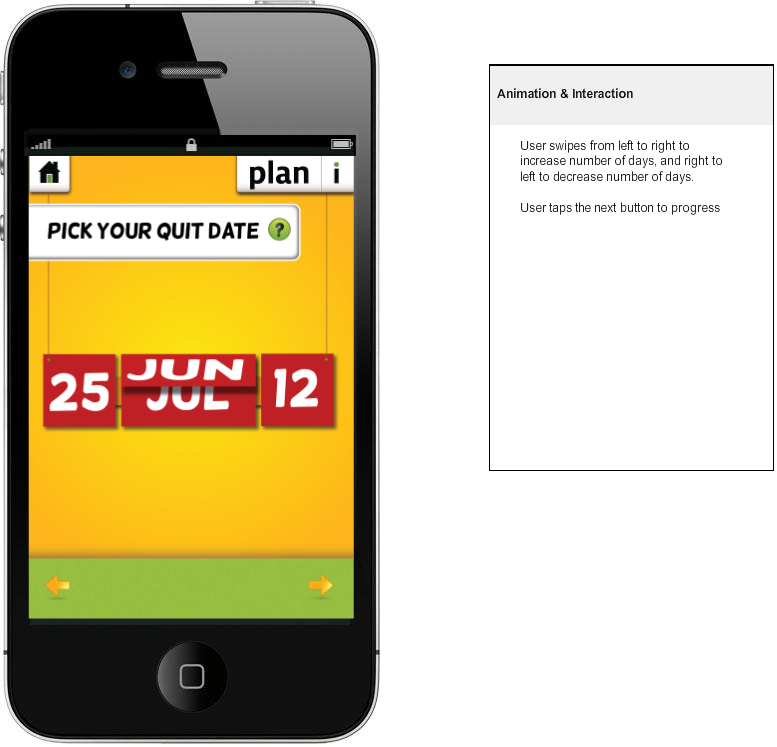


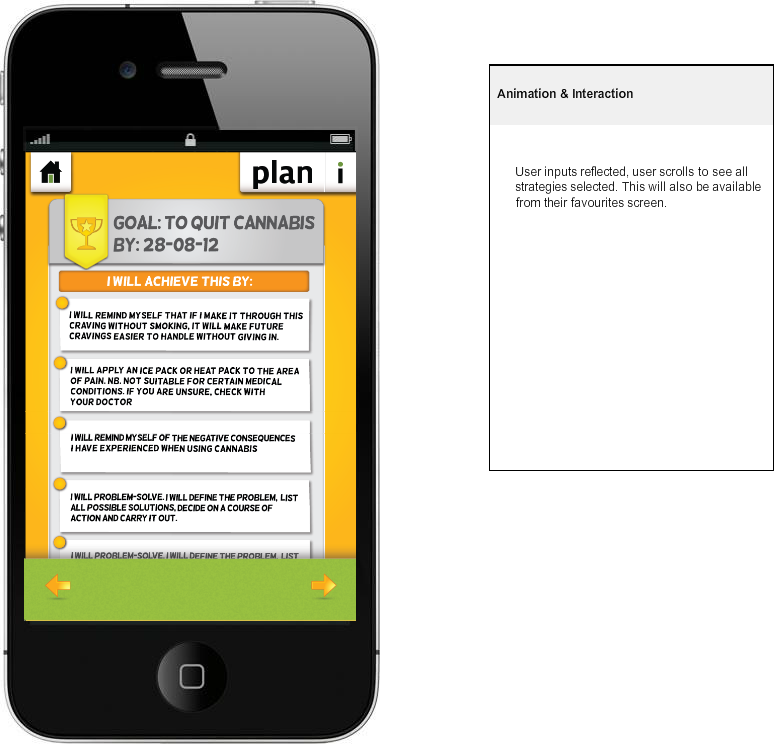

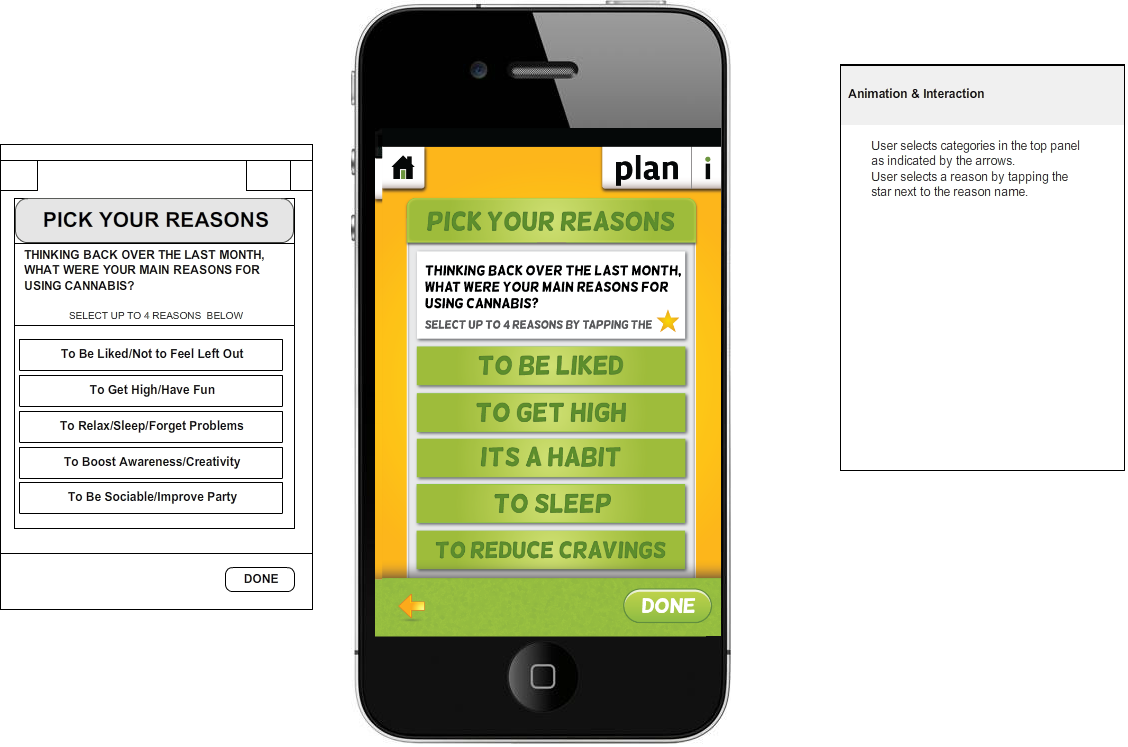

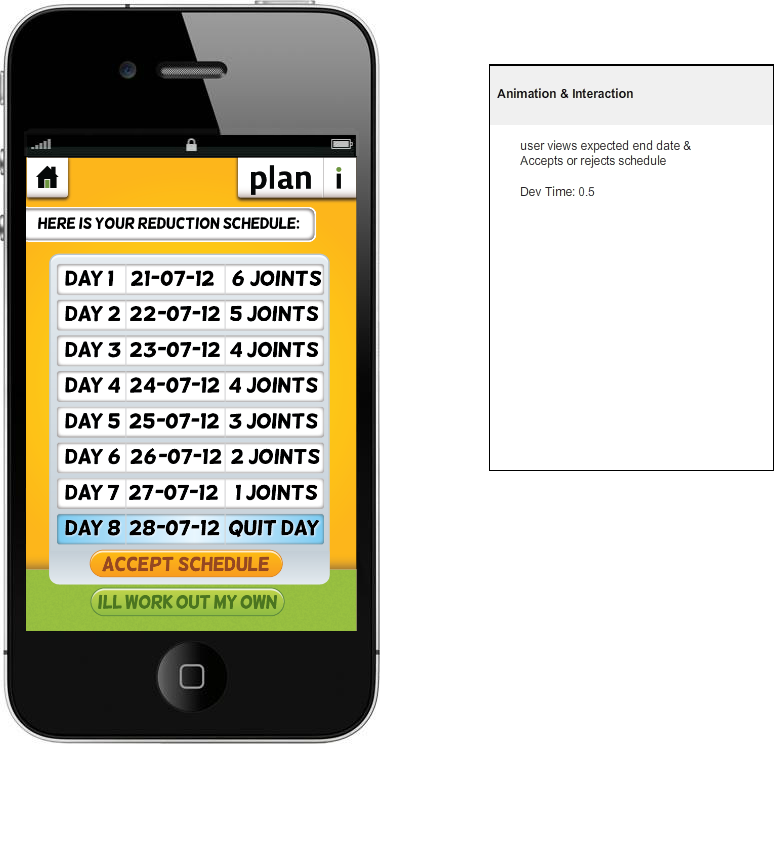


c) Track module


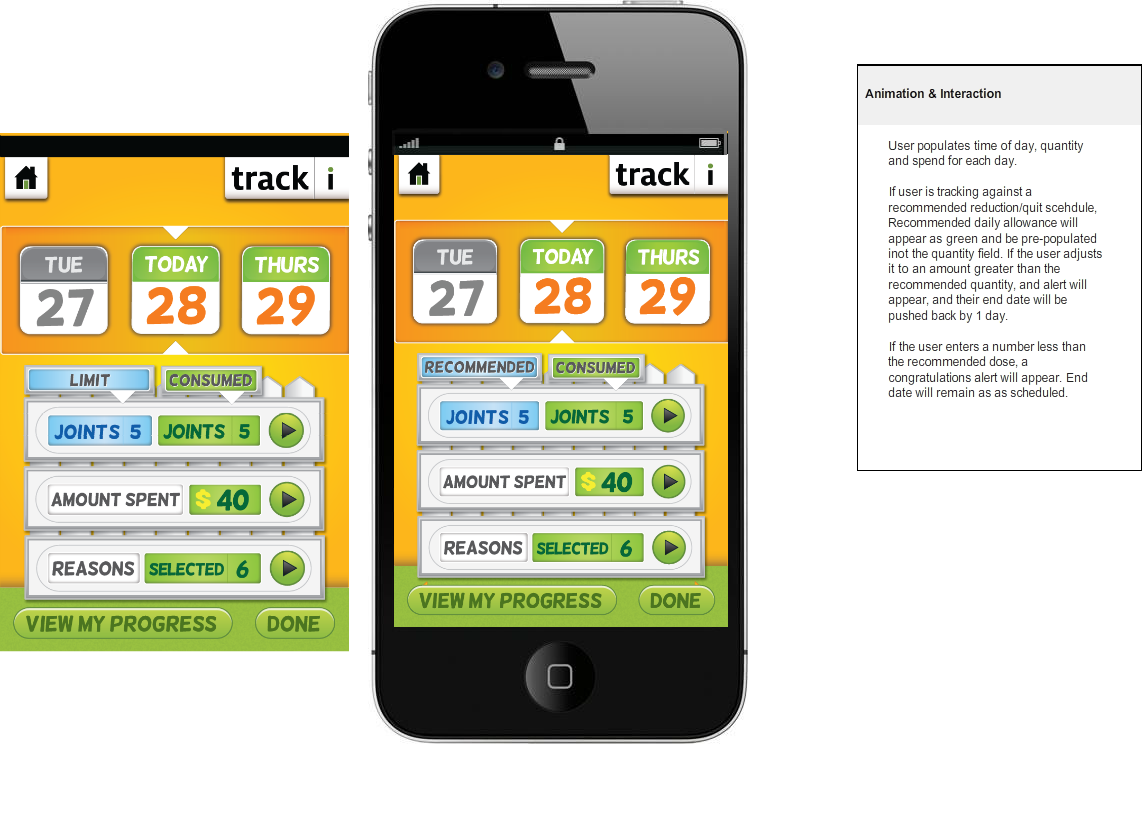

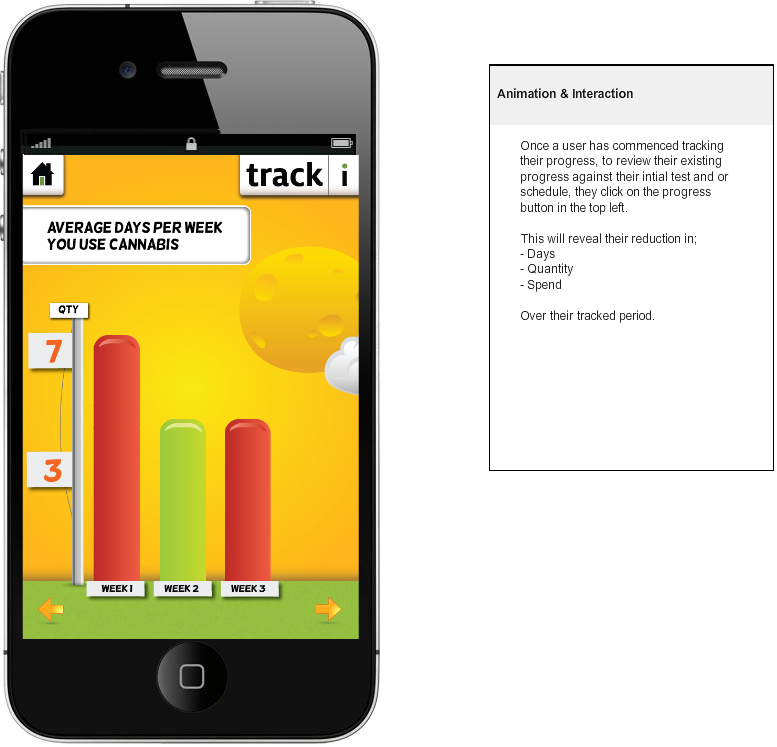

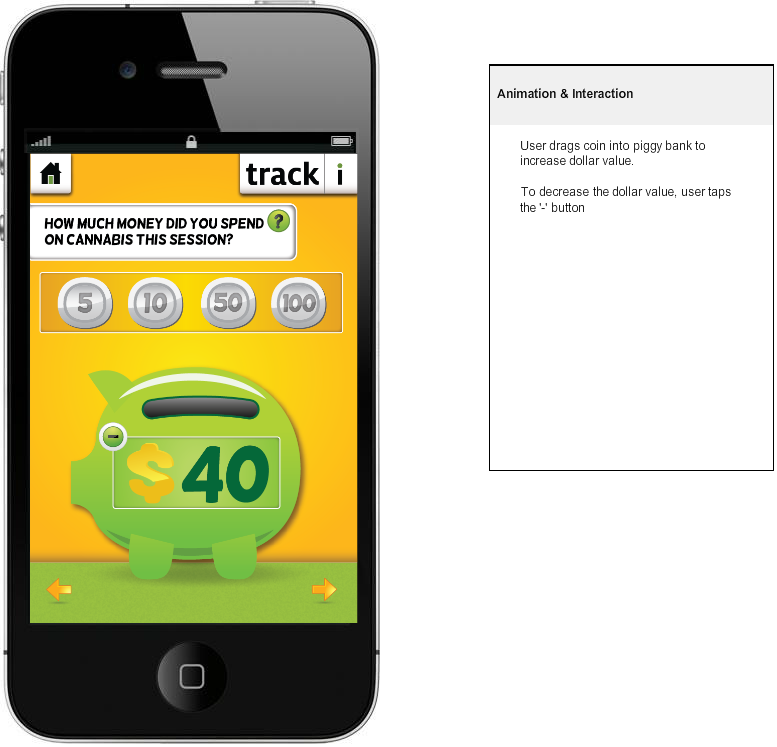

Supplement: Supplementary file 1 — Supplementary materials to accompany 'A Smartphone App Intervention for Adult Cannabis Users Wanting to Quit or Reduce Their Use: A Pilot Evaluation'. (DOCX 2601 kb) [file 42238_2019_9_MOESM1_ESM.docx]
